# Radiological Assessment of Different Retroperitoneal Lymph Node Measurements in Stage 1 Testicular Cancer Patients: Impact on Clinical Stage and Treatment

**DOI:** 10.3390/jcm13185553

**Published:** 2024-09-19

**Authors:** Angelina Strauch, Kai Nestler, Justine Schoch, Laura Kubitscheck, Stephan Waldeck, Hans Schmelz, Tim Nestler

**Affiliations:** 1 Department of Urology, Federal Armed Forces Hospital Koblenz, 56072 Koblenz, Germany; 2 Institute of Diagnostic and Interventional Radiology, Federal Armed Forces Hospital Koblenz, 56072 Koblenz, Germany

**Keywords:** germ cell tumor, testicular cancer, retroperitoneal lymph node metastasis, RECIST 1.1, staging imaging, lymph node measurement

## Abstract

**Background:** In staging for testicular germ cell tumor (GCT), current guidelines lack consensus regarding the measurement of retroperitoneal lymph node metastasis, concerning the recommended plane and dimension. This exploratory study aimed to assess its impact on clinical stage (cS) and therapy. **Methods:** We retrospectively examined 154 cSI (retroperitoneal lymph nodes < 10 mm in axial short-axis diameter (SAD)) GCT patients, without adjuvant therapy and a follow-up ≥ 24 months. Retroperitoneal lymph nodes were measured in staging images in different dimensions (SAD and long-axis diameter (LAD)) and planes (axial, sagittal and coronal). **Results:** Overall survival was 100%, with 82% free of recurrence after a median follow-up of 83 months. All patients were classified as cSI, based on axial SAD (RECIST 1.1). However, significantly more patients would have been classified as cSIIA (0% vs. 38% vs. 52%) or even cSIIB (0% vs. 1% vs. 25%) according to axial LAD (SWENOTECA, German S3 guideline) or maximum LAD in any plane (EAU, ESMO, AJCC and onkopedia) (*p <* 0.001). Overtreatment was predicted in 0%, 31% and 61% of patients based on axial SAD, axial LAD and maximum LAD, while undertreatment was estimated at 18%, 10% and 2%, respectively, (*p* < 0.001). **Conclusions:** These findings indicate considerable variability in cS based on current lymph node staging recommendations, suggesting that axial SAD (RECIST 1.1) could be the most appropriate parameter for standardized guideline recommendations.

## 1. Introduction

Testicular germ cell tumors (GCT) are the most common malignancy among young men between the ages of 20 and 40 years [1]. Although it is a very aggressive tumor entity, cure rates and overall survival of the mostly young patients have steadily improved for years due to advancements in treatment. However, long-term relative survival rate gradually decreases, even after 30 years of follow-up, likely due to the adverse effects of surgery, chemotherapy, or radiotherapy [2]. 

Staging in GCT patients to identify metastasis relies on cross-sectional imaging, which determines the clinical stage (cS) as follows: cSI indicates a non-metastasized tumor, cSII refers to retroperitoneal lymph node metastasis (IIA: ≥10–20 mm, IIB: >20–50 mm, IIC: >50 mm), and cSIII represents distant lymph node and organ metastasis [3,4,5,6,7]. The size of lymph nodes is consistently defined in current guidelines, but the specific dimensions—short-axis diameter (SAD) and long-axis diameter (LAD)—and planes (axial, sagittal or coronal), recommended for cross-sectional imaging, vary between sources [3,4,5,6,7,8]. The Response Evaluation Criteria In Solid Tumors 1.1 (RECIST 1.1), the radiological standard for monitoring treatment response in solid tumors, recommends using the maximum axial SAD [9]. In comparison, the maximum LAD in the axial plane is proposed by the S3-guideline of the German Society of Urology (DGU) [4]. Similarly, the Swedish and Norwegian Testicular Cancer Group (SWENOTECA) follows a “modified Royal Marsden Hospital staging system”, also based on axial LAD [8]. Most other guidelines, including those from the European Association of Urology (EAU) [3], the European Society for Medical Oncology (ESMO) [5], the American Joint Committee on Cancer (AJCC) [7] and onkopedia (Guidelines of the Medical Societies in Hematology and Medical Oncology of German speaking countries) [6], rely on the maximum LAD in any plane following the Tumor-Node-Metastasis (TNM) classification [10].

A recent survey of German urologists and genitourinary oncologists, specializing in the treatment of GCT patients, highlighted the clinical impact of these inconsistencies. Of the urologists surveyed, 55% used SAD in axial or any dimension, while 45% employed LAD, mostly in the axial dimension [11]. This variance could lead to discrepancies in staging (cS), which in turn affects treatment decisions, potentially leading to over- or undertreatment. Overtreatment could expose patients to unnecessary acute and long-term toxicities, while undertreatment may increase relapse risk. This is a very important issue, as most GCT patients are young with a long life expectancy. 

Therefore, the objective of this study was to compare the different lymph node measurement approaches at initial staging with consecutive cS and therapy, aiming to identify an optimal, standardized measurement parameter that balances the risks of over- and undertreatment while mitigating relapse risk.

## 2. Patients and Methods

### 2.1. Study Population

We retrospectively identified all GCT patients with cSI, diagnosed and treated at the Department of Urology, Federal Armed Forces Hospital in Koblenz, Germany, between 2000 and 2021 (*n* = 225). In our study, cSI was defined as retroperitoneal lymph nodes < 10 mm in axial SAD on computed tomography. Staging was performed at orchiectomy. Patients with suspiciously shaped lymph nodes, measuring <10 mm in axial SAD in the primary landing zone, or those with questionable cSIIA (in axial SAD), were re-staged after six weeks. They were only further examined, if marker-negative cSI was confirmed.

This exploratory study includes a total of 154 patients after orchiectomy without any adjuvant therapy, with normalized postoperative serum tumor markers, and a follow-up period ≥ 24 months. The entire patient inclusion/exclusion flowchart is depicted in Figure S1. We adhered to the Standards for Reporting of Diagnostic Accuracy Studies (STARD) recommendations [12].

Clinical data and pathological characteristics were assessed. The follow-up was conducted according to the recommendations of the German Testicular Cancer Study Group [4]. We defined relapse as enlargement of retroperitoneal lymph nodes ≥ 10 mm in axial SAD, elevation of tumor markers or distant metastasis during follow-up. We obtained ethical approval from the local ethics committee (2021-15756-retrospektiv).

### 2.2. Radiological Measurement

Two experienced uro-radiologists (KN, LK) independently measured retroperitoneal lymph nodes using the software Siemens Healthineers syngo.via VB60A. In case of inconsistent measurements concerning the largest lymph node in axial SAD, a consensus reading followed. The largest lymph node in axial SAD was subsequently measured across various dimensions (SAD and LAD) in the three different radiological planes: axial, sagittal and coronal.

We did not define a lower threshold value. If lymph nodes were too small to be precisely measured on 5-mm CT slices, a default value of 2.5 mm was applied for further statistical analysis of these lymph nodes.

### 2.3. Classification into Hypothetical Over-/Undertreatment

We also examined the differences in treatment resulting from the different lymph node measurement recommendations regarding relapse occurrence. Consequently, we concentrated on the threshold value for a pathological lymph node, defined as ≥10 mm according to current guidelines. 

Groups were defined as follows:

(1) correct staging/treatment
  (a) lymph node < 10 mm (≙cSI) and no relapse,
  (b) lymph node ≥ 10 mm (≙cS ≥ IIA) and relapse,
(2) overstaging/overtreatment = lymph node ≥ 10 mm (≙cS ≥ IIA) and no relapse,
(3) understaging/undertreatment = lymph node < 10 mm (≙cSI) and relapse.

In our study cohort, all patients were classified as cSI based on axial SAD and did not receive any adjuvant therapy. Thus, overstaging or overtreatment in axial SAD was absent. In comparison, according to the other lymph node measurements, which also included patients classified as cSI, we further analyzed the patients classified as hypothetical cS ≥ IIA, considering the need for the hypothetical adjuvant therapy they might have received.

Patients classified as “overtreated” would have undergone unnecessary radiotherapy or chemotherapy (30/36*Gy* or 3× BEP). “Undertreatment” would have led to delayed relapse diagnosis, potentially increasing cS and the International Germ Cell Cancer Collaborative Group (IGCCCG) risk group [13], thus necessitating more intense treatment—such as 36*Gy* instead of 30*Gy* for seminoma (if cSIIB instead of cSIIA) or 4× BEP instead of 3× BEP (if IGCCCG risk group would have been intermediate or poor).

### 2.4. Statistical Analysis

For statistical analysis, we used IBM *SPSS Statistics System* for Windows, v29.0 (Armonk, NY, USA). Categorical variables were presented as n (%), while continuous variables were reported as the median with 1st and 3rd quartiles. Group comparisons for continuous variables were performed using t-test, while categorial variables were analyzed using the Kruskal–Wallis test, Mann–Whitney test and Pearson‘s Chi-square test. A *p* value < 0.05 was considered statistically significant and a 95% confidence interval (CI) was applied. Effect sizes for mean differences between the different guideline recommendations on cS and therapy were calculated according to Cohen (1988) [14]. The classification was as follows: a small effect from r = 0.1, a medium effect from r = 0.3, and a large effect from r = 0.5.

In addition to the overall cohort analysis, we categorized patients into recurrence-free and those with recurrence during follow-up (≥24 months) after orchiectomy, to classify into hypothetical over- and undertreatment. Group comparisons were conducted using the aforementioned statistical tests. Binary logistic regression analysis was employed to determine associations between recurrence risk and histological risk factors. The corresponding effect sizes were reported using Cohen’s f^2^ (1988) [14], with a small effect from f^2^ = 0.02, a medium effect from f^2^ = 0.15, and a large effect from f^2^ = 0.35.

## 3. Results

### 3.1. Patient Characteristics

This study included a total of 154 cSI GCT patients. Seminoma and NSGCT (non-seminomatous germ cell tumor) were present in 106 (69%) and 48 (31%) patients, respectively. The majority had a low pT stage, with pT1 in 112 (73%) patients. Further patient characteristics are detailed in Table S1. Overall survival (OS) was 100% with a median follow-up of 83 (Q1 = 59; Q3 = 120) months. Relapse occurred in 27 (18%) patients, with a median relapse time of 14 (Q1 = 9; Q3 = 29) months, which is shown in the Kaplan–Meier curve in Figure S2. The relapse rates did not differ significantly between seminoma and NSGCT patients (*p* = 0.255; Figure S3). Most relapses were located in retroperitoneal lymph nodes (*n* = 25; 93%), while an exclusively mediastinal and inguinal lymph node relapse occurred in one patient (4%), respectively. Based on patients’ data, only initial pT stage was substantially higher in relapsed patients (*p* = 0.043; Table 1). At relapse, most patients (*n* = 19; 70%) presented with low-volume disease (cSIIA/B), and 26 (96%) patients were categorized as having a good prognosis according to the IGCCCG classification, with one patient (4%) classified as intermediate prognosis due to elevated serum tumor markers. No relapse occurred with isolated elevation of tumor markers. The most common treatment at relapse was 3–4× BEP (Bleomycin/Etoposide/Cisplatin) chemotherapy in 25 (93%) patients, while two (7%) patients received radiotherapy.

### 3.2. Influence on cS

**Figure 1:**
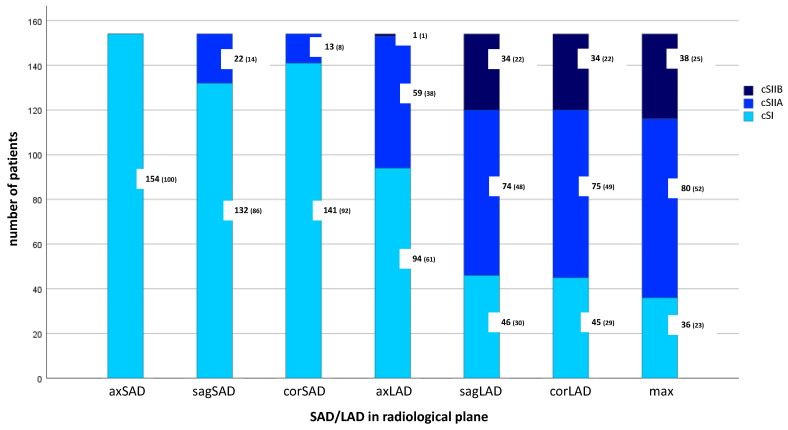
Clinical stages of the study cohort (*n* = 154) according to the different lymph node measurements (SAD/LAD in axial, sagittal and coronal plane), *p* < 0.001. Estimates were given as frequency (percentage). cS = clinical stage, axSAD = axial SAD, sagSAD = sagittal SAD, corSAD = coronal SAD, axLAD = axial LAD, sagLAD = sagittal LAD, corLAD = coronal LAD, max = maximum LAD in any plane.

cS differed significantly depending on the radiological retroperitoneal lymph node measurement recommended by different guidelines (*p* < 0.001; Figure 1). According to axial SAD measurement (RECIST 1.1), all patients were classified as cSI. In contrast, according to SWENOTECA and DGU, which recommend the axial LAD, only 94 (61%, CI [53.9, 68.2]) patients would be classified as cSI. Other guidelines, using the maximum LAD, would result in just 36 (23%, CI [17.5, 29.9]) patients being classified as cSI, with 80 (52%, CI [44.7, 59.1]) patients classified as cSIIA and even 38 (25%, CI [18.8, 30.7]) patients as cSIIB.

The discrepancy between RECIST 1.1 and SWENOTECA or DGU indicated a medium effect size based on Cohen’s criteria (*p* < 0.001, r = 0.49). A medium effect size was also shown for the comparison between SWENOTECA or DGU and the other guidelines (such as EAU, ESMO, onkopedia, AJCC) (*p* < 0.001, r = 0.44). However, the difference between RECIST 1.1 and the other guidelines demonstrated a large effect size according to Cohen (*p* < 0.001, r = 0.77). 

### 3.3. Influence on Therapy

The differing lymph node measurement methods recommended by current guidelines would also result in significantly different therapeutic recommendations (*p* < 0.001; Table S2). Using axial SAD (RECIST 1.1), 120 (78%, CI [72.1, 83.8]) patients were classified as cSI without risk factors, which led to a surveillance-based treatment. The remaining 34 (22%, CI [16.2, 27.9]) patients were classified as cSI with risk factors. They had the choice between surveillance and adjuvant therapy (1× BEP or 1× Carboplatin AUC 7), with all choosing surveillance. However, based on axial LAD (SWENOTECA, DGU) and maximum LAD (other guidelines), the number of cSI patients without risk factors with recommendation for surveillance would be only *n* = 77 (50%, CI [42.9, 57.6]) and *n* = 29 (19%, CI [13, 24.7]), respectively. In axial LAD, only 17 (11%, CI [7.1, 14.9]) patients, and in maximum LAD, only 7 (5%, CI [1.9, 6.5]) patients would be classified as cSI with risk factors. Substantially more patients would be diagnosed as metastasized with cSIIA/B (*n* = 60; 39%, CI [32.5, 46.1] and *n* = 118; 77%, CI [69.7, 83.1]) and therefore would be treated with 3× BEP, or for seminoma alternatively with 30/36*Gy* radiotherapy. In summary, stage-appropriate therapy would hypothetically require 34 optional cycles of chemotherapy (BEP or Carboplatin) for axial SAD (RECIST 1.1) compared to 361 for maximum LAD (other guidelines) (Table S2). 

Again, the differences between RECIST 1.1 and SWENOTECA or DGU and SWENOTECA or DGU and the other guidelines presented a medium effect size according to Cohen (*p* < 0.001, r = 0.37, respectively). Meanwhile, the difference between RECIST 1.1 and the other guidelines showed a large effect size according to Cohen (*p* < 0.001, r = 0.72).

### 3.4. Influence on Over-/Undertreatment

**Figure 2:**
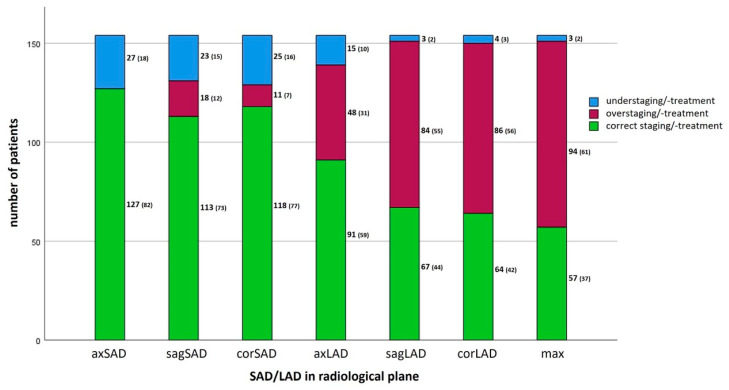
Categorization of the study cohort (*n* = 154) in the 3 different groups “correct staging/treatment”, “overstaging/overtreatment” and “understaging/undertreatment” according to the different lymph node measurements (SAD and LAD in axial, sagittal and coronal plane), *p* < 0.001. Estimates were given as frequency (percentage). axSAD = axial SAD, sagSAD = sagittal SAD, corSAD = coronal SAD, axLAD = axial LAD, sagLAD = sagittal LAD, corLAD = coronal LAD, max = maximum LAD in any plane.

Significant differences were observed across the three groups (correct staging and correct treatment, overstaging/overtreatment, understaging/undertreatment) depending on the different lymph node measurements (*p* < 0.001), as summarized in Figure 2. By using axial SAD, most patients would be treated appropriately (*n* = 127; 82%, CI [76.9, 87.7]). In contrast, based on maximum LAD, the fewest patients would be treated correctly (*n* = 57; 37%, CI [30.5, 44.2]). Overtreatment would occur in axial SAD, axial LAD and maximum LAD in 0%; 31%, CI [24.7, 38.3] and 61%, CI [53.9, 67.5] (*p* < 0.001), while undertreatment would affect 18%, CI [12.3, 22.7]; 10%, CI [5.8, 14.3] and 2%, CI [0, 4.5] of the patients, respectively, (*p* < 0.001).

### 3.5. Association between Histological Risk Factors and Relapse

We examined the histological risk factors such as tumor size and rete testis invasion in seminoma patients, while LVI was assessed in NSGCT patients. We compared patients with and without relapse. Among seminoma patients, only tumor size was significantly larger in those who experienced relapse compared to those who remained relapse-free (*p* = 0.026). The other parameters did not show substantial differences. 

In binary logistic regression analysis, a significant correlation was observed between tumor size and relapse in seminoma patients (*p* = 0.031). For each unit increase in tumor size, the relative relapse probability increased by 3.3%, with a weak effect size (Cohen’s f^2^ = 0.09). No significant association was found between LVI and relapse risk in NSGCT patients.

## 4. Discussion

Our study population was representative regarding OS, age, time to relapse and histology [15,16,17]. Only relapse rate in NSGCT was lower than expected compared to other studies with 13% instead of the anticipated 15–50% [15,18]. Regarding seminoma, the 20% relapse rate fell in the expected range of 5–30% [19,20,21]. In NSGCT, histological risk factors were less present (LVI = 13%) than in seminoma patients (rete testis infiltration and testicular tumor size = 26%), which might explain the lower relapse rate in the NSGCT cohort.

According to current guideline recommendations, the number of cS ≥IIA cases and overtreatment was considerably higher when measuring maximum LAD, as proposed by EAU, ESMO, AJCC and onkopedia, or maximum LAD in axial plane, as recommended by SWENOTECA and DGU, compared to SAD in axial plane, as recommended by RECIST 1.1. “Undertreated” patients with a delayed relapse diagnosis, mostly based on SAD measurement, could still be adequately treated with similar survival rates [20,22]. In the event of a higher clinical stage at delayed relapse diagnosis, these patients would only potentially receive a higher toxicity of chemo- or radiotherapy (one more cycle of BEP, if IGCCCG ≥ intermediate, or 36*Gy* instead of 30*Gy*, only in seminoma if cSIIB instead of cSIIA). In contrast, initial false-positive patients, mainly based on LAD measurement, would be treated with unnecessary chemotherapy (at least 3× BEP) or radiotherapy (30*Gy* or 36*Gy*) [3,4,5,6,7].

To date, this is the first study to examine the differences in cS and treatment based on the inconsistent staging recommendations across various guidelines. Therefore, we propose standardizing guidelines, to provide the best possible ratio between over- and underdiagnosis, balancing the risk between unnecessary acute and long-term therapeutic toxicity and relapse for the mostly young GCT patients [23]. Given the OS rate of 100% in our cohort, the axial SAD, as recommended by RECIST 1.1, might be the most appropriate parameter for lymph node staging, as it resulted in the lowest rate of overtreatment without increasing the rate of recurrence. 

RECIST 1.1 is the established radiological guideline in other oncological diseases for the evaluation of lymph nodes, leading to consistent diagnostic and therapeutic results [9,24]. While it does not seem applicable to treatment monitoring of new targeted tumor therapies, it remains the standard recommendation for staging and follow-up of conventional chemo- and radiotherapeutic therapies [25]. In addition, using an already established radiological parameter could be more suitable for hospitals that are not specialized in treating GCT. 

The threshold value ≥ 10 mm in axial SAD is also commonly used for primary lymph node staging in various other oncological diseases, and in men’s most common tumors: prostate-, lung- and colorectal cancer. However, its diagnostic reliability is well discussed due to its low sensitivity [26,27,28]. For diagnostic enhancement in these tumors, other criteria in addition to the lymph node size, such as radioligand imaging, were adapted and are nowadays standardly used [29,30]. However, the therapeutic consequences of cross-sectional imaging differ substantially between the aforementioned tumors and GCT. While those tumors predominantly refer to histopathological assessment of regional lymph nodes that have been resected in primary surgery to determine possible adjuvant therapy, GCT depends on accurately analyzing retroperitoneal lymph node metastasis in cross-sectional imaging for staging and treatment [3,4,5,6,7].

Yet, cross-sectional imaging as a diagnostic tool has its limitations in accuracy for staging. Therefore, further approaches for adequate risk stratification and individualized therapy for patients have been established. For cSI, there are well-known histological risk factors: for seminoma tumor size > 4 cm and rete testis invasion; for NSGCT LVI. Our results revealed a significant association only between tumor size and the occurrence of relapse in seminoma patients, suggesting that established risk factors provide only an approximation of optimal relapse prediction. Consequently, recent studies have sought to refine prognostic models by incorporating additional factors to better stratify patients and once again, reduce overtreatment [31,32]. Furthermore, the combination of radiological staging and serum tumor markers might enhance diagnostic reliability of GCT [33]. 

Since conventional serum tumor markers have only limited sensitivity and specificity, extensive research into new tumor markers for GCT has been conducted in recent decades. A relatively new approach is measuring microRNA (miR)-371a-3p levels as a biomarker in serum. Its sensitivity and specificity are superior to conventional serum tumor markers (>90% vs. 50%) and it correlates strongly with tumor burden and cS of GCT (except teratoma) [34,35]. Furthermore, it is a promising diagnostic tool concerning the early detection of recurrence during follow-up of GCT patients [35,36]. However, it is not yet established in clinical practice. Another attempt to reduce overtreatment in marker-negative NSGCT cSIIA patients is the re-staging imaging after six weeks. According to the current EAU guideline, GCT patients should be treated like cSI, if the lymph node metastases decrease. If the metastases remain stable, further staging should be completed after six weeks; if the metastases remain unchanged, primary retroperitoneal lymph node dissection (RPLND) should be performed. Chemotherapy should be applied if the tumor markers are positive or if the tumor progresses to cSIIB [3]. Moreover, in patients with low-volume metastatic cSIIA/B seminoma, ongoing research into primary RPLND seeks to spare patients the need for chemotherapy [37].

Staging imaging in GCT can also be enhanced through Radiomics, a machine learning algorithm that predicts lymph node metastasis by analyzing large imaging databases [38]. In particular, the combined “radiomics-clinical model,” which incorporates additional clinical factors, appears to outperform “radiomics-only” prediction models [38]. It should be noted that those Radiomics studies focusing on initial staging imaging in GCT, lack histological confirmation of lymph node metastasis. Nevertheless, a study evaluating Radiomics for predicting malignant retroperitoneal lymph nodes prior to RPLND in NSGCT, which included histological validation, also highlighted Radiomics as a promising diagnostic tool [39].

Remarkably, some GCT studies fail to provide specific definitions of the planes and dimensions employed for their radiological lymph node measurements, which were used for clinically staging their study cohort [15,17,19,34]. This lack of standardization in study protocols raises doubts about the overall comparability of prior GCT studies at specified cS. Differing lymph node size criteria in other oncological studies were also the main reason to implement RECIST 1.1 as a standardized reference, which has already demonstrated its value by ensuring consistent diagnosis, treatment and comparability in clinical trials for patients across different studies [24].

Adapting staging criteria in international guidelines also has ethical implications. On the one hand, understaging may lead to preventable harm for the patients, due to an insufficient initial evaluation of their disease, compromising their prognosis and well-being. On the other hand, overstaging may result in overtreatment by causing avoidable harm when the disease could have been managed with less aggressive approaches. In general, a physician should discuss all benefits and risks of different treatment options, so patients are properly informed and can accept or refuse any recommended medical treatment [40]. As understaging in GCT patients is very likely to result in similar survival rates [20,22], we regard it as the preferable option for these patients. However, borderline cases, such as patients with an axial SAD of 10 mm ± 2 mm, could be monitored more closely with earlier follow-up imaging, similar to the approach used for NSGCT patients with questionable cSI. 

Our study’s retrospective design and the limited sample size (*n* = 154) present certain limitations. Therefore, this study should be viewed primarily as hypothesis-generating, providing a basis for further research. A prospective multicenter study with a larger study cohort, corresponding to a robust power analysis, is essential to draw definitive conclusions for clinical practice and, ultimately, to contribute to the adaptation of international guidelines. 

Due to our inclusion criteria, axial SAD < 10 mm as maximum for the lymph node size and no adjuvant therapy after orchiectomy, the study cohort is possibly biased in favor of patients with an overall better prognosis. Possibly, patients with generally “smaller” and therefore more benign lymph nodes were included. Moreover, some patients with risk factors who received adjuvant therapy after initial staging were excluded. This would predominantly apply to NSGCT patients as they have a higher risk of recurrence at cSI than seminoma patients. These factors might have influenced our findings regarding relapse rates, particularly in relation to our limited cohort size. However, these inclusion criteria should be maintained in future prospective studies, though patient selection biases would be minimized in such a design.

Yet, our cohort is contemporary and representative, because a variety of different lymph node sizes was analyzed. We even included patients with lymph nodes ≥10 mm in cross-sectional imaging in all measurements but the axial SAD measurement. Additionally, most relapses were diagnosed only radiologically and not by histological evaluation, as is commonplace in clinical practice.

## 5. Conclusions

Our study reveals that inconsistency in current guidelines leads to very different clinical stages and treatment recommendations in GCT patients. There is an urgent need for lymph node measurement standardization to reduce avoidable acute and long-term therapeutic toxicity. Considering the OS rate of 100% and a non-elevated relapse rate in our cohort, using axial SAD as recommended by RECIST 1.1 resulted in the lowest rate of overtreatment. Consequently, this method may represent the best approach for a consistent lymph node staging. As our study was only exploratory, standardization of guideline recommendations requires further investigation through a prospective multicenter study.

## Figures and Tables

**Table 1:** Clinical data of the study cohort (*n* = 154), comparison of patients with and without relapse. Estimates were given as median (quartile 1, quartile 3) or frequency (percentage), *p* values were given based on Mann–Whitney-, Pearson’s Chi-square- and *t*-test.

Clinical Parameters	Patients without Relapse	Patients with Relapse	*p*
number of patients, *n* (%)	127 (82)	27 (18)	
patient age at diagnosis (years)			0.834
median (quartile 1, quartile 3)	35 (29, 42)	39 (26, 44)	
tumor histology, *n* (%)			0.269
seminoma	85 (67)	21 (78)	
non-seminoma	42 (33)	6 (22)	
embryonal cell carcinoma	28 (22)	5 (19)	
teratoma	26 (20)	0	
choriocarcinoma	10 (8)	0	
yolk sac tumor	14 (11)	2 (7)	
seminoma	11 (9)	2 (7)	
pT-stage, *n* (%)			0.043
1	96 (76)	16 (59)	
2	22 (17)	9 (33)	
3	2 (2)	1 (4)	
4	0	0	
unknown	7 (6)	1 (4)	
testicular tumor size (mm)			0.053
median (quartile 1, quartile 3)	28 (18, 40)	35 (20, 60)	
infiltration of rete testis, *n* (%) unknown	23 (18) 14 (11)	9 (33) 4 (15)	0.139
lymphovascular invasion, *n* (%)			
pL	16 (13)	5 (19)	0.648
pV	16 (13)	6 (22)	0.40
unknown	14 (11)	2 (7)	
tumor marker nadir median (quartile 1, quartile 3)			
AFP (norm < 5.8 IU/mL)	2.4 (1.8, 3.3)	2.7 (2.1, 4.4)	0.413
ß-hCG (norm < 5 mIU/mL)	0.7 (0.3, 1.4)	0.4 (0, 1.3)	0.216
LDH (norm < 250 U/L)	173 (152, 203)	177 (154, 194)	0.913
follow-up (months)			0.326
median (quartile 1, quartile 3)	85 (56, 125)	81 (62, 89)	

AFP = alpha fetoprotein, ß-hCG = human choriongonadotropin, LDH = lactate dehydrogenase.

## Data Availability

The data of this study are available from the corresponding author upon reasonable request.

## Supplementary Materials

The following supporting information can be downloaded at: https://www.mdpi.com/article/10.3390/jcm13185553/s1, Figure S1: The flowchart summarizes patient accrual according to STARD recommendations. Figure S2: Kaplan–Meier survival curve of the entire study cohort (*n* = 154) with a minimum follow-up of 27 months and a maximum of 286 months. Figure S3: Kaplan–Meier survival curve of the study cohort separated by seminoma (*n* = 106) (blue line) and NSGCT (*n* = 48) (red line) patients. Table S1: Clinical parameters of the study cohort (*n* = 154). Estimates were given as median (quartile 1, quartile 3) or frequency (percentage). Table S2: Treatment recommendations for the study cohort (*n* = 154) depending on the different lymph node measurements/guidelines.
