# Supplementary material for: Radiological Assessment of Different Retroperitoneal Lymph Node Measurements in Stage 1 Testicular Cancer Patients: Impact on Clinical Stage and Treatment
Source: J Clin Med. 2024 Sep 19;13(18):5553. doi: 10.3390/jcm13185553 (PMC11432456; doi:10.3390/jcm13185553)
Supplement: Supplementary file 1 [file jcm-13-05553-s001.zip › jcm-3183731-supplementary.pdf]

Journal of Clinical Medicine

MDPI

Supplementary Information:

Radiological Assessment of Different Retroperitoneal Lymph Node Measurements in Stage 1 Testicular Cancer Patients: Impact on Clinical Stage and Treatment

Angelina Strauch [1,†], Kai Nestler [2,†], Justine Schoch [1], Laura Kubitscheck [2], Stephan Waldeck [2], Hans Schmelz [1] and Tim Nestler [1,*]

[1] Department of Urology, Federal Armed Forces Hospital Koblenz, 56072 Koblenz, Germany
[2] Institute of Diagnostic and Interventional Radiology, Federal Armed Forces Hospital Koblenz, 56072 Koblenz, Germany
[*] Correspondence: tim-nestler@web.de
[†] These authors contributed equally to this work.

Figures:

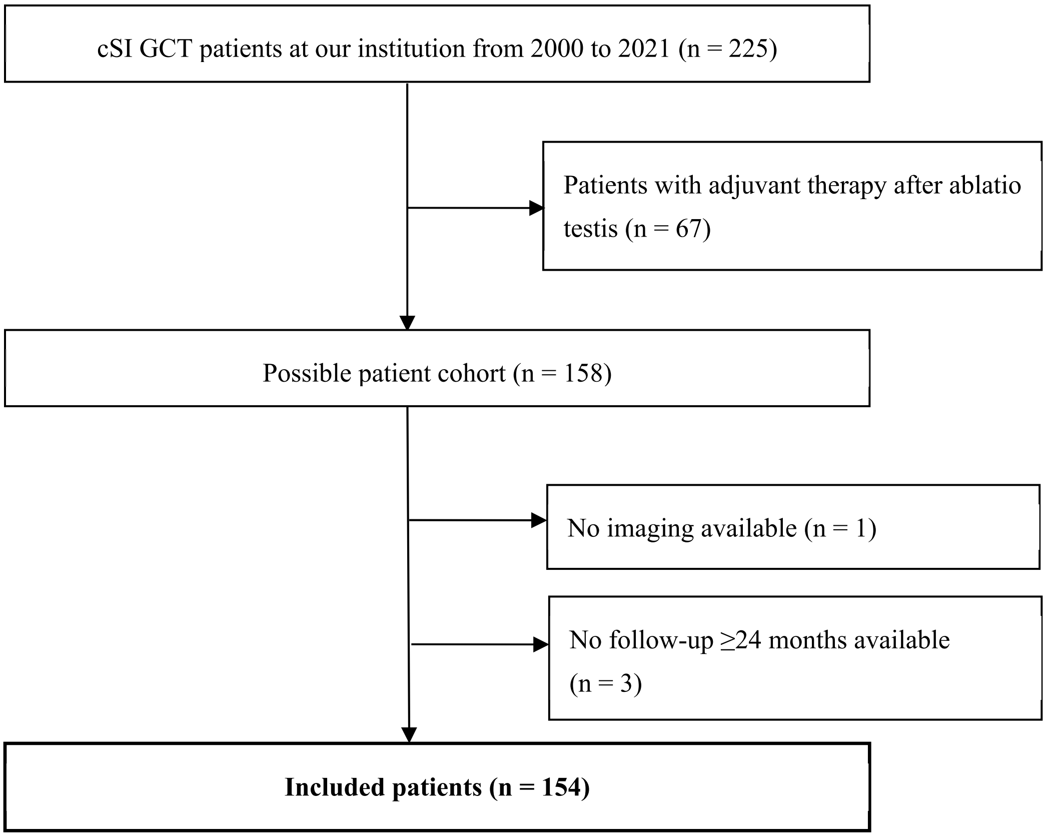

Figure S1. The flowchart summarizes patient accrual according to STARD recommendations. cS = clinical stage, GCT = testicular germ cell tumor

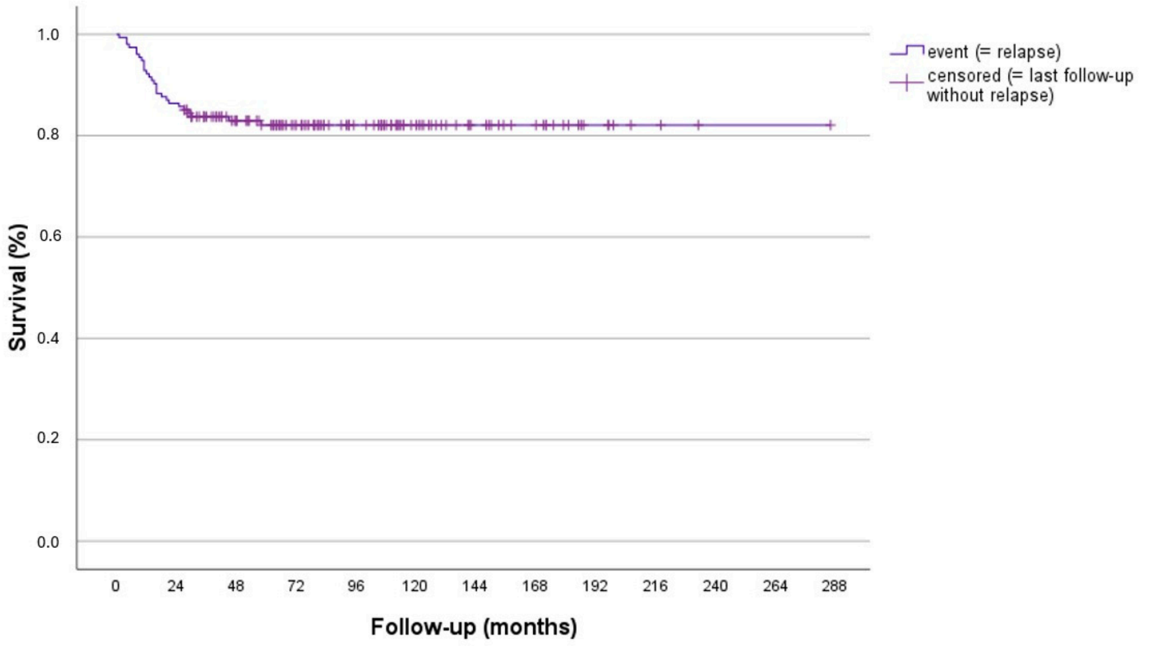

Figure S2. Kaplan-Meier survival curve of the entire study cohort (n = 154) with a minimum follow-up of 27 months and a maximum of 286 months.

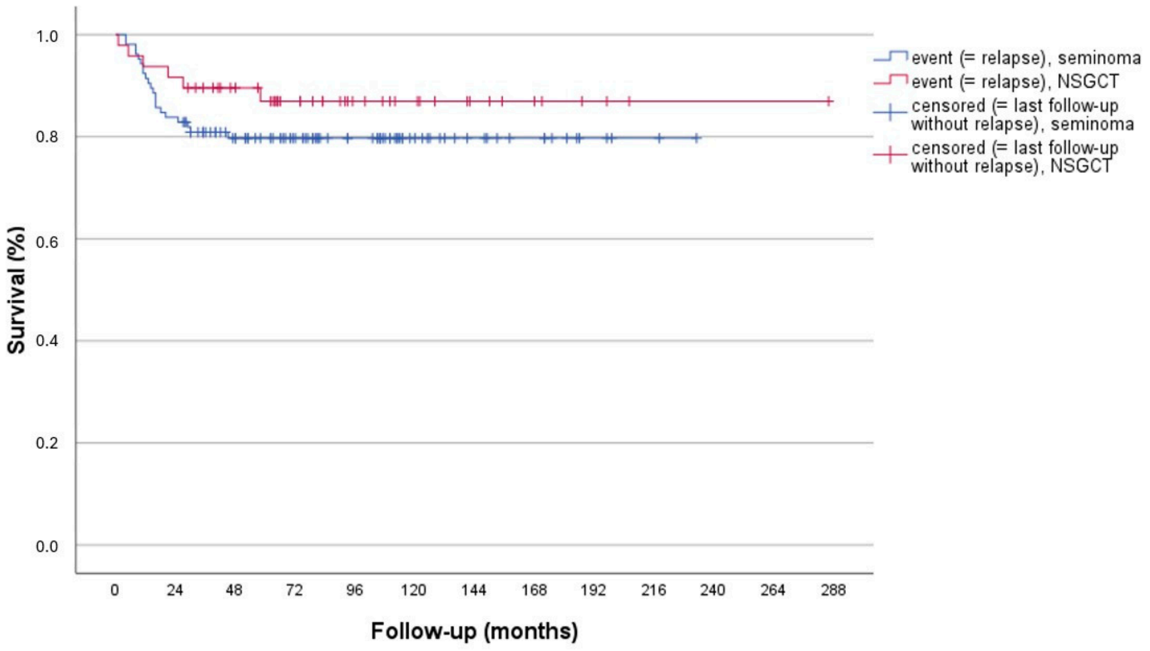

Figure S3. Kaplan-Meier survival curve of the study cohort separated by seminoma (n = 106) (blue line) and NSGCT (n = 48) (red line) patients. NSGCT = non-seminomatous germ cell tumor

Tables:

Table S1. Clinical parameters of the study cohort (n = 154). Estimates were given as median (quartile 1, quartile 3) or frequency (percentage).

**clinical parameters**	
**number of patients, n (%)**	154 (100)
**patient age at diagnosis (years)**	
median (quartile 1, quartile 3)	35 (28, 43)
**tumor histology, n (%)**	
seminoma	106 (69)
non-seminoma	48 (31)
embryonal cell carcinoma	33 (69)
teratoma	26 (54)
choriocarcinoma	10 (20)
yolk sac tumor	16 (33)
seminoma	13 (27)
**pT-stage, n (%)**	
1	112 (73)
2	31 (20)
3	3 (2)
4	0
unknown	8 (5)
**testicular tumor size (mm)**	
median (quartile 1, quartile 3)	30 (18, 43)
**infiltration of rete testis, n (%)**	32 (21)

unknown	18 (12)
**lymphovascular invasion, n (%)**	29 (19)
pL	21 (14)
pV	22 (14)
unknown	16 (10)
**tumor marker nadir**	
**median (quartile 1, quartile 3)**	
AFP (norm <5.8 IU/ml)	2.5 (1.9, 3.4)
ß-hCG (norm <5 mIU/ml)	0.6 (0.3, 1.4)
LDH (norm <250 U/l)	173 (152, 201)
**follow-up (months)**	
median (quartile 1, quartile 3)	83 (59, 120)
**relapse, n (%)**	27 (18)
**deaths**	0

AFP = alpha fetoprotein, ß-hCG = human choriongonadotropin, LDH = lactate dehydrogenase

Table S2. Treatment recommendations for the study cohort (n = 154) depending on the different lymph node measurements/guidelines, $p < 0.001$. The colors stand for the toxicity of the therapies: green – surveillance, yellow – surveillance or 1 cycle of Carboplatin AUC 7/BEP, red – 3 cycles of BEP or 30/36*Gy* radiotherapy. Estimates were given as frequency (percentage).

cS and histology		axSAD	sagSAD	corSAD	axLAD	sagLAD	corLAD	max	therapy
**cSI**	seminoma + RF	28 (18)	20 (13)	24 (16)	13 (8)	5 (3)	4 (3)	4 (3)	surveillance/ 1x Carboplatin
	NSGCT + RF	6 (4)	5 (3)	5 (3)	4 (3)	3 (2)	3 (2)	3 (2)	surveillance/ 1x BEP
	without RF	120 (78)	107 (69)	112 (73)	77 (50)	38 (25)	38 (25)	29 (19)	surveillance
**cSIIA**	seminoma	0	18 (12)	8 (5)	43 (28)	55 (36)	55 (36)	61 (40)	3x BEP/30*Gy*
	NSGCT	0	4 (3)	5 (3)	16 (10)	19 (12)	20 (13)	18 (12)	3x BEP
**cSIIB**	seminoma	0	0	0	0	18 (12)	20 (13)	21 (14)	3x BEP/36*Gy*
	NSGCT	0	0	0	1 (1)	16 (10)	14 (9)	18 (12)	3x BEP
**guidelines**		RECIST 1.1			DGU SWENOTECA			others	

cS = clinical stage, axSAD = axial SAD, sagSAD = sagittal SAD, corSAD = coronal SAD, axLAD = axial LAD, sagLAD = sagittal LAD, corLAD = coronal LAD, max = maximum LAD in any plane, NSGCT = non-seminomatous germ cell tumor, RF = risk factor, BEP = Bleomycin/Etoposide/Cisplatin, RECIST 1.1 = Response Evaluation Criteria In Solid Tumors 1.1, DGU = S3 guideline of the German Society of Urology, SWENOTECA = Swedish and Norwegian Testicular Cancer Group, others = other guidelines (EAU = European Association of Urology, onkopedia = Guidelines of the Medical Societies in Hematology and Medical Oncology of the German speaking countries, AJCC = American Joint Committee on Cancer, ESMO = European Society for Medical Oncology)
